# Sex differences in anxiety and depression in children with attention deficit hyperactivity disorder: Investigating genetic liability and comorbidity

**DOI:** 10.1002/ajmg.b.32842

**Published:** 2021-05-03

**Authors:** Joanna Martin, Sharifah Shameem Agha, Olga Eyre, Lucy Riglin, Kate Langley, Leon Hubbard, Evie Stergiakouli, Psychiatric Genomics Consortium ADHD Working Group, Michael O'Donovan, Anita Thapar

**Affiliations:** 1 MRC Centre for Neuropsychiatric Genetics and Genomics, Division of Psychological Medicine and Clinical Neurosciences, Cardiff University, Cardiff, UK; 2 Cwm Taf Morgannwg University Health Board Health Board, Wales, UK; 3 School of Psychology, Cardiff University, Cardiff, UK; 4 MRC Integrative Epidemiology Unit, University of Bristol, Bristol, UK; 5 Population Health Sciences, University of Bristol, Bristol, UK

**Keywords:** ADHD, ALSPAC, anxiety disorders, depression, polygenic risk scores, sex differences

## Abstract

It is unknown why attention deficit hyperactivity disorder (ADHD) is more common in males, whereas anxiety and depression show a female population excess. We tested the hypothesis that anxiety and depression risk alleles manifest as ADHD in males. We also tested whether anxiety and depression in children with ADHD show a different etiology to typical anxiety and depression and whether this differs by sex. The primary clinical ADHD sample consisted of 885 (14% female) children. Psychiatric symptoms were assessed using standardized interviews. Polygenic risk scores (PRS) were derived using large genetic studies. Replication samples included independent clinical ADHD samples (*N* = 3,794; 25.7% female) and broadly defined population ADHD samples (*N* = 995; 33.4% female). We did not identify sex differences in anxiety or depression PRS in children with ADHD. In the primary sample, anxiety PRS were associated with social and generalized anxiety in males, with evidence of a sex-by-PRS interaction for social anxiety. These results did not replicate in the broadly defined ADHD sample. Depression PRS were not associated with comorbid depression symptoms. The results suggest that anxiety and depression genetic risks are not more likely to lead to ADHD in males. Also, the evidence for shared etiology between anxiety symptoms in those with ADHD and typical anxiety was weak and needs replication.

## 1 Introduction

Attention deficit hyperactivity disorder (ADHD) is a common and highly heritable neurodevelopmental disorder (Thapar, 2018). Neurodevelopmental disorders, including ADHD and autism spectrum disorder (ASD), are typified by a male excess in prevalence (Thapar, Cooper, & Rutter, 2017). In childhood, ADHD is 2–7 times more frequently diagnosed in males than females, though by adulthood the rate is similar in males and females (Franke et al., 2018). The male excess in ADHD prevalence in childhood is especially prominent in clinically ascertained samples but it is also present in community samples (Faraone et al., 2015). The reasons why ADHD is more common in males in childhood are not yet known (Rutter, Caspi, & Moffitt, 2003).

In contrast, depression and anxiety, which share genetic liability and commonly co-occur with ADHD, are more frequently diagnosed in females in adolescence and adulthood and the reasons are also unknown (Craske et al., 2017; Dalsgaard et al., 2019; Faraone et al., 2015; Faraone & Larsson, 2018; Malhi & Mann, 2018; Martin, Taylor, & Lichtenstein, 2018; Wray et al., 2018). Given these sex differences in prevalence, one hypothesis is that the same alleles that confer risk for ADHD and neurodevelopmental disorders in males, may manifest as other psychiatric problems, such as anxiety and depression, in females. Recent genetic and family studies support this hypothesis by demonstrating that genetic and familial risks for ADHD and neurodevelopmental disorders (i.e., family history, common polygenic variation, or large, rare copy number variants) may be more strongly associated with anxiety and depression in females than in males (Kendall et al., 2018; Martin, Tammimies, Karlsson, et al., 2018; Martin, Taylor, Rydell, et al., 2018; Martin et al., 2020).

A related hypothesis, that could help explain the lower diagnosis rates of anxiety and depression observed in males, is that alleles which confer risk for anxiety/depression manifest as ADHD and neurodevelopmental disorders in males. This would mean that males diagnosed with ADHD may carry a higher burden of risk alleles that are shared with anxiety and depression compared with females with ADHD. To our knowledge, this has not been examined to date in the context of a clinical ADHD sample.

Individuals with neurodevelopmental disorders, including ADHD, also show elevated rates of comorbid anxiety and depression (Eyre et al., 2019; Faraone et al., 2015). These comorbid problems are often more common in females than in males with ADHD (Quinn, 2008), as is the case in the general population (Craske et al., 2017; Malhi & Mann, 2018). Although there is some evidence of high genetic correlations (*r*_*g*_ > 0.7) between “internalizing” problems in childhood and diagnosed anxiety and depression in adults (Jami et al., 2020), these correlations were examined in the context of psychiatric symptoms in children in the general population. An unresolved question is whether comorbid anxiety and depression in individuals with ADHD are aetiologically similar to typical anxiety and depression in non-ADHD samples and whether this differs by sex.

The primary aim of this study was to test the hypothesis that males with ADHD have a higher genetic liability to anxiety and depression than females with ADHD. As an exploratory test, we also determined whether there are sex differences in genetic risk for other major psychiatric disorders in those with ADHD, given that genetic liabilities for disorders such as schizophrenia also confer risk for anxiety and depression (Lee et al., 2019; Purves et al., 2019). The second aim was to test whether the burden of risk alleles for anxiety and depression is associated with symptoms of anxiety and depression (respectively) in individuals with ADHD and whether there are any sex differences in this association. As anxiety and depression in those with ADHD could arise as a result of ADHD itself (Riglin et al., 2020), we also assessed whether comorbid anxiety and depression were associated with ADHD risk alleles.

## 2 Method

### 2.1 Attention deficit hyperactivity disorder sample

Children and adolescents (aged 5–18 years) with a confirmed or suspected clinical diagnosis of ADHD were recruited through Child and Adolescent Psychiatry or Pediatric out-patient clinics across the United Kingdom, primarily in Wales. Approval for the study was obtained from the North West England and Wales Multicenter Research Ethics Committees, as well as the Cardiff University School of Medicine Research Ethics Committee. Written informed consent to participate was obtained from all parents and from adolescents aged 16–18 years old and assent was gained from children under 16 years of age.

Children were originally included in the study if they met DSM-III-R or DSM-IV research-based diagnostic criteria for ADHD, confirmed using the parent version of the Child and Adolescent Psychiatric Assessment (CAPA) (Angold et al., 1995) a semi-structured diagnostic interview. Parents were asked about the presence of the nine inattentive and nine hyperactive–impulsive symptoms from the DSM-IV and two additional symptoms used only in the DSM-III-R. Impairment and onset of symptoms was also assessed. Pervasiveness of symptoms was confirmed using teacher reports [Child ADHD Teacher Telephone Interview (ChATTI) (Holmes et al., 2004), and Conners’ Teacher Rating Scale (Conners, Sitarenios, Parker, & Epstein, 1998)]. CAPA interviews were undertaken by trained psychologists and cases were supervised weekly by an experienced child and adolescent psychiatrist (Professor Anita Thapar). Interrater reliability for ADHD diagnosis and subtype, assessed using 60 cases, was perfect (*κ* = 1.0). Children were included if they met ADHD diagnostic criteria according to DSM-IV, with a small subset (*N* = 117) from an earlier part of the study who met DSM-III-R criteria; also, a small subset of children (*N* = 24) met lifetime criteria but no longer had sufficient symptoms to meet the diagnostic criteria at the time of assessment; analyses without these children were run as a sensitivity check (see below). A total of *N* = 1,114 children (mean age = 10.5 years, *SD* = 2.8) with ADHD were recruited, of whom *N* = 162 (14.5%) were female.

Symptoms of comorbid anxiety and depression in the preceding 3 months were also assessed using the parent-reported CAPA, according to DSM-IV symptom criteria (see Table S1 for details of the symptoms). The CAPA asks about anxiety symptoms including Separation Anxiety Disorder of Childhood, Generalized Anxiety Disorder (GAD), and Social Phobia/Social Anxiety Disorder. It also includes a section on Depression (see Table S1 for details). The rate of DSM-IV anxiety and depression diagnoses was very low in this sample (Cooper, Martin, Langley, Hamshere, & Thapar, 2014). The CAPA is best suited for obtaining categorical/diagnostic information and not generating continuous traits. Thus, binary variables were derived to indicate presence of one or more anxiety symptoms for each of the three anxiety subtypes and also for any anxiety symptoms, as well as presence of a core depression symptom (depressed mood or anhedonia) or any depression symptoms (see Table S1). Children and adolescents aged 12 and older also completed the Child version of the CAPA. A symptom was considered as present if either the parent or child reported it.

Socioeconomic and cognitive measures were also completed and were used to compare males and females with ADHD. Family annual income, parental employment status, and parental educational attainment were assessed by parental questionnaire. Low income was defined as annual family income less than £20,000 (equivalent ~US $32,000), and parental low educational attainment was defined as having left school without qualifications (GCSE or equivalent) at age 16 years. Socioeconomic status (SES) was classified by the occupation of the main family wage earner using the UK Standard Occupation Classification (Office of National Statistics, 2001). Two SES categories were defined (low: unskilled workers/unemployed; medium/high: manual and nonmanual skilled/partially skilled workers, and professional and managerial workers). Full-scale IQ was assessed using the Wechsler Intelligence Scale for Children (WISC), version III or IV, using all 10 subtests (Wechsler, 1992, 2003).

### 2.2 Genetic data

DNA samples (from saliva or blood) were collected and genotyped in several batches, with rigorous quality control procedures (see Supporting Information Text). A total of 3,335,041 SNPs and 885 children with ADHD (13.9% female) of European ancestry passed all quality control and were included in the analyses.

Polygenic risk scores (PRS) for common autosomal variants were derived in PLINK based on six large psychiatric disorder discovery GWAS of primarily European ancestry, with no overlap with the target sample: anxiety disorders (31,977 cases and 82,114 controls) (Purves et al., 2019), major depressive disorder (MDD; 59,851 cases and 113,154 controls) (Wray et al., 2018), ADHD (18,378 cases and 29,113 controls) (Demontis et al., 2019), schizophrenia (67,390 cases and 94,015 controls) (Schizophrenia Working Group of the Psychiatric Genomics Consortium, 2020), autism spectrum disorder (ASD; 18,382 cases and 27,969 controls) (Grove et al., 2019), and bipolar disorder (BD; 20,352 cases and 31,358 controls) (Stahl et al., 2019). PRS were calculated using LD-clumping in PLINK using seven different *p*-value thresholds. The first principal component was extracted and analyzed based on the correlation matrix for these different PRS, using the PRS–PCA approach (Coombes, Ploner, Bergen, & Biernacka, 2020; see details in Supporting Information Text).

### 2.3 Analyses

Prior to testing our main aims, we first examined whether there were phenotypic differences in males and females with ADHD, by testing for sex differences in socioeconomic and clinical characteristics.

To address the first aim, we tested for sex differences in anxiety and depression PRS. We also explored sex differences in PRS for ADHD, schizophrenia, bipolar disorder and ASD, to determine whether there are any other notable sex-specific genetic effects.

To address the second aim, we tested for associations between anxiety and depression PRS with the presence of anxiety and depression symptoms (respectively) in the whole sample, and also stratified by sex (conservative Bonferroni corrected *p*-value threshold of .0083; based on four variables related to anxiety and two variables related to depression symptoms). We then tested for PRS-by-sex interactions. We also explored whether ADHD risk allele burden was associated with presence of anxiety and depression symptoms.

For all analyses, females were coded as “1” and males were coded as “0.” The top 5 ancestry-based PCs (in line with previous work using samples with <1,000 individuals; Demontis et al., 2019) and genotyping batch were included as covariates in all PRS analyses. The sample included 47 families with full-siblings and half-siblings. Given these nonindependent genetically-related observations, we specified family clusters and applied a sandwich estimator to estimate cluster-robust standard errors of regression coefficients. All analyses used generalized estimating equations implemented in the *drgee* package in R. Nagelkerke *R*^2^ differences between null and full models were calculated to obtain estimates of variance explained.

### 2.4 Sensitivity analyses

Given the wide age range of the sample (5–18 years old) and young mean age (10.5 years), as a sensitivity analysis, we stratified the sample using the mean age at assessment and repeated the analyses for both groups (younger: 5–10 years and older: 11–18 years). To account for potential differences in those with DSM-III-R ADHD and only lifetime ADHD, the main analyses were also repeated excluding children who did not meet DSM-IV ADHD criteria at the time of assessment (*N* = 108 with genetic data excluded).

### 2.5 Replication samples

We also used a larger dataset of independent ADHD samples from the Psychiatric Genomics Consortium (PGC) (Demontis et al., 2019) as a replication sample, to test our main study hypothesis. This consisted of nine European ancestry PGC studies that did not overlap with our primary ADHD sample (totaling 974 female ADHD cases and 2,820 male ADHD cases); the samples were primarily of children and adolescents with ADHD, with three of the studies including adults (samples from Bergen, Spain, and Yale-Penn). They were included to maximize the sample size, given evidence of a high genetic correlation between child and adult ADHD (Rovira et al., 2020). PRS for anxiety and depression were derived using the same method as above, ensuring no overlap between discovery GWAS and target samples. Analyses were run on each of the nine studies separately and the results were meta-analyzed using a fixed effects model implemented in the *metafor* package in R, and weighted mean variance explained was calculated. Secondary phenotypic data for presence of anxiety and depression symptoms were not available in this sample.

We also used a general population sample of children, the Avon Longitudinal Study of Parents and Children (ALSPAC). ALSPAC is a large, well-characterized longitudinal study (Boyd et al., 2013; Fraser et al., 2013). See Supporting Information Text for details of the sample, including phenotypic definitions and genetic data. Analyses were limited to a group of children with broadly defined likely ADHD problems, defined as anyone who met the parent-rated Strengths and Difficulties Questionnaire (SDQ) (Goodman, 1997) hyperactivity subscale cut-point at least once between the ages of 4–13 years (using data from six time points). Anxiety was assessed using the Development and Well-Being Assessment (DAWBA), parent-rated at age 7–13 and self-rated at age 15 years (Goodman, Ford, Richards, Gatward, & Meltzer, 2000). Binary variables were derived to indicate presence of any anxiety symptoms between the ages of 7–15 years and also for the three subtypes of anxiety (GAD, separation anxiety, and social phobia). PRS were derived using the same method as above. A total of 995 children (12.7% of the sample, 33.4% female) who passed genetic quality control met criteria for broadly defined ADHD. The rate of anxiety symptoms in those with broadly defined ADHD was compared with the rest of the ALSPAC sample. PRS analyses included only the broadly defined ADHD group.

## 3 Results

The primary sample of children diagnosed with ADHD included 123 females and 762 males. Females and males were similar in terms of socioeconomic factors, IQ, ADHD diagnosis subtype and symptoms, and presence of anxiety and depression symptoms (see Table 1).

### 3.1 Sex differences in polygenic risk scores (within attention deficit hyperactivity disorder cases)

We did not detect sex differences in anxiety or depression PRS in our primary sample (see Table 2). Analyses of the larger (*N* = 3,794, 25.7% female) PGC ADHD replication sample showed weak evidence of higher anxiety PRS in females than males [OR(CIs) = 1.09(1.00–1.18), *p* = .040, *R*^2^ = 3.9 × 10^−3^], but little evidence for a sex difference in depression PRS [OR(CIs) = 1.04(0.95–1.13), *p* = .38, *R*^2^ = 1.8 × 10^−3^; see Figure S1]. Using the broadly defined ADHD group in ALSPAC, there was little evidence for sex differences in anxiety PRS [OR (CIs) = 1.06(0.93–1.21), *p* = .41, *R*^2^ = 9.5 × 10^−4^] or depression PRS [OR(CIs) = 1.07(0.94–1.22), *p* = .31, *R*^2^ = 1.5 × 10^−3^]. The exploratory analyses of PRS for other major psychiatric disorders also showed little evidence for sex differences in the primary clinical ADHD sample (Table 2).

### 3.2 Association of polygenic risk scores with co-occurring anxiety or depression symptoms

In the primary ADHD sample, we found that anxiety PRS were associated with presence of GAD [OR(CIs) = 1.38(1.09–1.76), *p* = .0082, *R*^2^ = 0.017] and social anxiety symptoms [OR(CIs) = 1.38(1.06–1.81), *p* = .017, *R*^2^ = 0.017], though the latter association results did not survive correction for multiple testing; see Figure and Table S2 for detailed results. Sex-stratified analyses revealed associations between anxiety PRS and GAD [OR(CIs) = 1.52(1.16–2.00), *p* = .0026, *R*^2^ = 0.028] and social anxiety symptoms [OR(CIs) = 1.54(1.15–2.06), *p* = .0040, *R*^2^ = 0.029] in males; there was also weak evidence of association with any anxiety symptoms, that did not survive multiple testing correction [OR(CIs) = 1.21(1.02–1.43), *p* = .028, *R*^2^ = 0.011]. There was little evidence of association in females (*p* > .01; see Table S2). The sex-by-PRS interaction analysis indicated a moderating effect of sex on the relationship between anxiety PRS and presence of social anxiety symptoms (*p* = .0049) and also any anxiety symptoms (*p* = .0055) in the children with ADHD, with stronger associations observed in males than in females.

**Figure 1:**
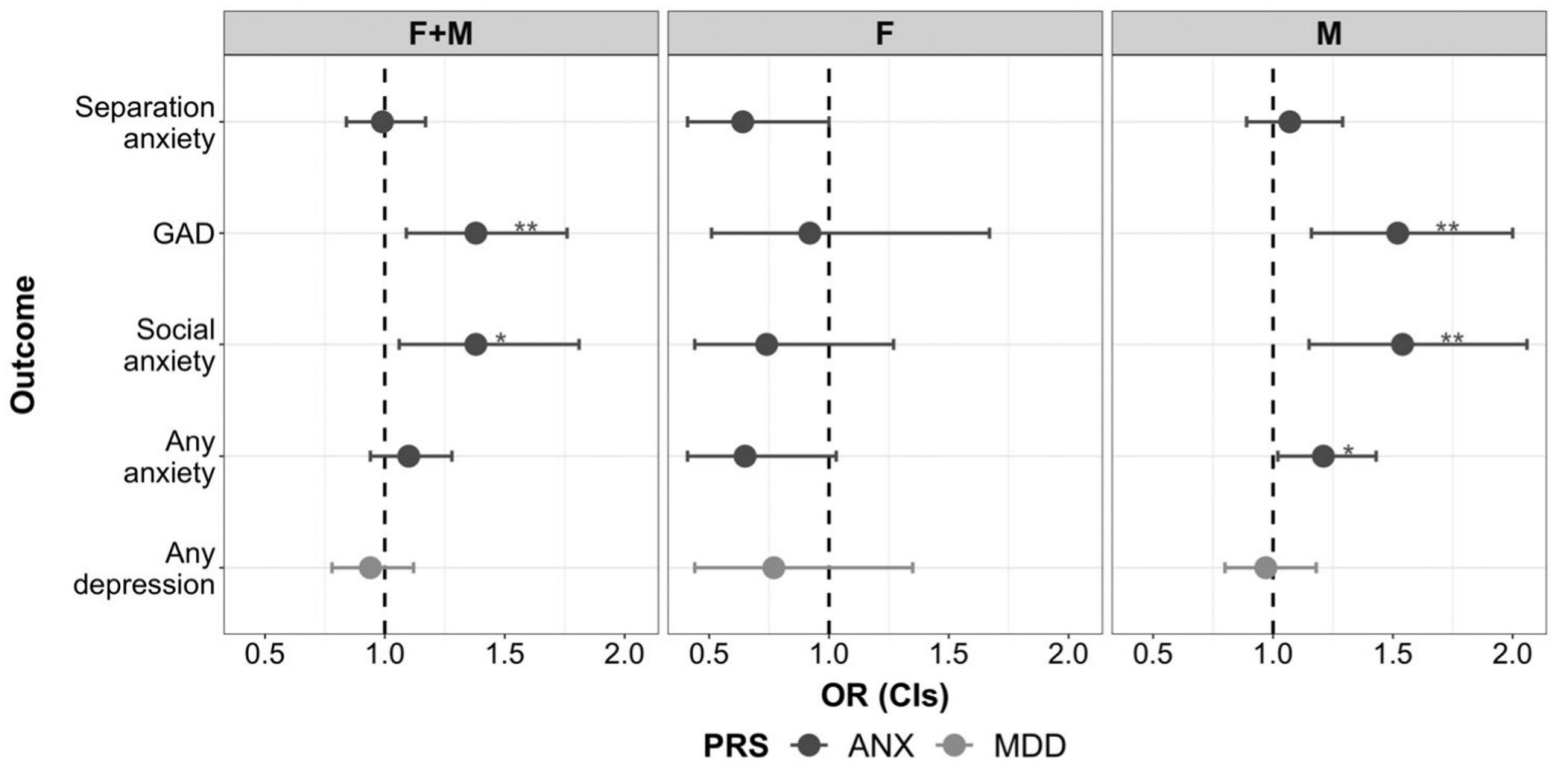
Results of the association between polygenic risk scores (PRS) for anxiety disorders (ANX) and major depressive disorder (MDD) and presence of symptoms of anxiety and depression (respectively) in the primary clinical sample of children with ADHD. Results are presented for the whole sample (females + males; F + M) and for females (F) and males (M) separately. * *p* < .05, ** *p* < .01 (associated after Bonferroni correction for multiple testing). GAD, generalized anxiety disorder

There was little evidence of association between depression PRS and presence of depressive symptoms either in the whole sample or in the sex-stratified sample (see Figure 1; Table S2). Given the high rate (75.7%) of any depression symptoms in the sample, we also repeated the analysis using the two “core” diagnostic criteria of depression (depressed/irritable mood or loss of pleasure; rate: 10.5%) to focus on a more stringent phenotype and found a similar pattern of results (Table S2).

In the group of children with broadly defined ADHD in ALSPAC, 29.8% were classed as having GAD symptoms, 12.1% as having social anxiety symptoms, and 14.4% as having separation anxiety symptoms. Children with broadly defined ADHD were more likely to have any anxiety compared with those without ADHD [41.9 vs. 27.8%; OR(CIs) = 1.88(1.54–2.29), *p* = 6.2 × 10^−10^]. In the ADHD group, girls were more likely than boys to have any anxiety [48.3 vs. 38.4%; OR(CIs) = 1.50(1.03–2.18), *p* = .035]. There was little evidence of an association between anxiety PRS and anxiety symptoms in the group of individuals with broadly defined ADHD in ALSPAC (*N* = 995, 33.4% female), or that this association differed for males and females, thus not replicating the results in the ADHD clinical sample (Table S3).

Our exploratory analyses revealed little evidence of association between ADHD PRS and co-occurring symptoms of anxiety or depression in the primary clinical ADHD sample (Table S4).

### 3.3 Sensitivity analyses

In the primary clinical ADHD sample, there were 470 children aged between 5 and 10 years and 415 children aged between 11 and 18 year at assessment. The stratified analyses showed a similar pattern of results to the main analyses (Tables S5 and S6), with largely overlapping confidence intervals in the two age groups. Analyses excluding children in the primary clinical ADHD sample who did not meet DSM-IV ADHD criteria at the time of assessment (*N* = 108 excluded) showed a similar pattern of results to the main analyses (Tables S7 and S8).

## 4 Discussion

In this study of children with ADHD, we tested for sex differences in polygenic burden for depression and anxiety. The results did not support our hypothesis of a higher polygenic burden for anxiety and depression in males with ADHD compared with affected females. We did find some evidence that anxiety PRS are associated with co-occurring symptoms of anxiety in children diagnosed with ADHD, as well as evidence that this association is stronger in males than females, but these results did not extend to a broader definition of ADHD in children in the general population. Contrary to previous studies (Quinn, 2008), we found no sex differences in prevalence of comorbid anxiety and depression symptoms, though this may have been owing to the small number of females, young age of the sample (mean age = 10.5 years), and use of symptoms rather than diagnoses. We note that the rates of anxiety and depression are known to increase from adolescence onward (Dalsgaard et al., 2019).

Genetic risk for ADHD and other neurodevelopmental disorders appears to be associated with anxiety and depression in females, primarily in children with clinical diagnoses, but also to some extent in adults (Kendall et al., 2018; Martin et al., 2020; Martin, Tammimies, Karlsson, et al., 2018; Martin, Taylor, Rydell, et al., 2018). Given this, we set out to test the converse, that is, to determine whether anxiety and depression risk alleles are enriched in males with ADHD compared with affected females. Using three independent ADHD datasets, we did not find support for this hypothesis. Indeed, there was weak evidence of slightly higher anxiety PRS observed in females (compared with males) with ADHD in the larger PGC ADHD sample. There are several potential interpretations of these results. It may be that our hypothesis is incorrect, and that risk alleles that are shared across ADHD and anxiety/depression do not manifest in a sex-specific manner. Alternatively, if the same genetic risks do manifest in a sex-specific manner, the impact of anxiety/depression common risk alleles could be smaller than the impact of ADHD risk alleles and thus more difficult to detect. This is plausible, given that anxiety and depression are only moderately heritable (anxiety: 30–60%; depression: 31–42%), compared with the higher heritability of ADHD (70–80%), which impacts on the power of GWAS of these disorders and the amount of variance that can be explained by common variants (Faraone & Larsson, 2018; Hettema, Neale, & Kendler, 2001; Sullivan, Neale, & Kendler, 2000). In any case, our results suggest that there are no substantial, or clinically meaningful cross-disorder polygenic burden sex differences in individuals with diagnosed ADHD, or in those with broadly defined ADHD in the population. Previous studies reported no sex differences in ADHD polygenic burden in children with ADHD (Martin, Taylor, Rydell, et al., 2018; Martin, Walters, Demontis, et al., 2018). Our study adds to this work, finding a similar polygenic burden for other major psychiatric disorders (i.e., anxiety, MDD, ASD, bipolar disorder, and schizophrenia) in males and females with ADHD. These results indirectly imply that sex differences in the manifestation of shared common risk alleles are unlikely to explain why anxiety and depression are less common in the general population in males or that this is because these risks are manifesting as ADHD or other neurodevelopmental disorders. They also do not support a sex-specific liability threshold model (sometimes referred to as the female protective effect) of ADHD (Martin, Walters, Demontis, et al., 2018; Taylor et al., 2016).

We found no association of MDD PRS with presence of co-occurring clinical symptoms of depression in children with diagnosed ADHD. This is consistent with other studies that did not find an association between MDD PRS and mood symptoms related to depression (i.e., irritability, sadness or emotional dysregulation) in children with diagnosed ADHD (Nigg et al., 2020; Riglin et al., 2017). It also supports studies that suggest that depression in individuals with ADHD may be a consequence of the difficulties of having ADHD (Riglin et al., 2020; Stern et al., 2020). Building on these studies, we also found no evidence of association between ADHD genetic risk and depression symptoms in the context of an existing diagnosis of ADHD. Several issues need to be considered in interpreting this lack of association between MDD PRS and depression symptoms. First, the majority of the UK clinical ADHD sample is young (mean age was 10.5 years) and had not passed through the typical risk period for depression, which is after mid-adolescence; the analyses stratified by age at assessment also do not support an association between MDD PRS and depression symptoms in the older sub-group, though they are still a childhood sample (Table S6). Second, the definition of depression we used was broad, considering the presence of any symptoms in childhood, with the majority of the ADHD sample (75.7%) having at least one symptom. Third, the MDD GWAS was based on clinical diagnoses in adults and MDD may differ in etiology from depression in children, as suggested by several studies (Musliner et al., 2019; Rice et al., 2019; Riglin et al., 2020; Thapar & McGuffin, 1994; Thapar & Riglin, 2020). Indeed a recent GWAS of internalizing symptoms in children in the general population found a genetic correlation of approximately 0.7 with adult anxiety and MDD GWAS, suggesting a moderately high degree of shared common variant effects but also some specificity (Jami et al., 2020). Fourth, there could also be phenotypic differences between symptoms of depression in the context of ADHD compared with non-ADHD samples, which could partly explain the null results. Thus although we found little evidence of association between MDD PRS and depression symptoms in children with ADHD, we cannot conclude whether this is due to different etiology of depression in the context of ADHD per se or other factors, such as the use of sub-threshold definitions of depression or differences in etiology across adult and child depression. Future studies should also examine comorbid diagnoses in adults with ADHD.

In contrast to the result for depression, we found that the anxiety PRS were associated with presence of anxiety symptoms (particularly social anxiety and GAD) in the clinical ADHD sample. A sex-by-PRS interaction analysis showed that the association between anxiety PRS and social anxiety symptoms was stronger in males than females. However, caution is required in interpretation due to the small sample size of the female group and also because the results were not replicated using the broader definition of ADHD symptoms in an independent general population sample of children. This lack of replication may be due to the differences between these samples, such as differences in assessment measures and age of assessment, and the fact that the broadly defined ADHD group will have included individuals who do not experience impairment from their ADHD or comorbid anxiety symptoms, or have transient symptoms. We can conclude that our findings do not extend to a broader definition of ADHD and further work is needed using clinical ADHD samples to determine whether our preliminary findings are robust.

In addition to the issues discussed above, this study has several strengths and limitations. A key strength of the primary ADHD sample is that this group of children is representative of children with ADHD seen in clinics in Wales and the United Kingdom. They are severely affected and impaired, with cognitive difficulties and clinical comorbidities. One limitation is that the primary clinical ADHD sample was mainly prepubertal in age and as this was a cross-sectional study, we were unable to consider developmental changes in childhood and adolescence or lifetime risk of anxiety/depression. The PGC replication sample, although better powered for analyses related to a primary diagnosis of ADHD, lacked data on secondary phenotypes such as comorbid anxiety and depressive symptoms. Also, a notable exception to the representativeness of the ADHD samples is that owing to the limitations of PRS common variant genomic approaches (Martin et al., 2019), individuals of non-European ancestry were not included in analyses. Larger clinical samples of children with ADHD from diverse populations and with information on comorbid symptoms are needed to confirm the results of this study. We were not able to separate genetic risks into those that are shared between ADHD, anxiety, and depression, versus those that are unique to each disorder or indeed shared with other psychiatric disorders more broadly; future studies would benefit from examining the relative impact of these different genetic risk categories. A final issue related to within case analyses is that the sample is already enriched for ADHD risk alleles and other neuropsychiatric liabilities that are known to correlate with ADHD genetic risk. This means that very large samples are needed to detect differences within cases.

In conclusion, our findings did not support sex differences in anxiety or depression polygenic burden in children with ADHD. This suggests that genetic risks shared across ADHD and anxiety/depression are not manifesting as ADHD in males, which indirectly implies that the female excess of anxiety and depression in the population may not be explained by cross-disorder shared genetic effects. We also did not find robust evidence of association between anxiety and depression common risk alleles and comorbid symptoms of anxiety and depression in children with ADHD, with only weak evidence for an association for anxiety in clinically diagnosed males. Thus, we are unable to confidently conclude whether the etiology of comorbid anxiety and depression in the presence of ADHD differs from typical clinically diagnosed anxiety and depression. This is an important area of further study as it will help to inform clinicians on whether standard treatments are likely to be effective for anxiety and depression in the context of childhood ADHD.

## Supplementary Material

SM

Appendix

## Figures and Tables

**Table 1:** Characteristics of males and females with ADHD

Phenotype	Females (*N* = 123)		Males (*N* = 762)		Statistics	
	*N* (%)		*N* (%)		OR (LCI-UCI)	*p*
Socioeconomic status						
Low	53 (52.0)		323 (49.1)		1.12 (0.74–1.70)	.59
Medium-high	49 (48.0)		335 (50.9)			
Family income						
Low	54 (64.3)		281 (62.7)		1.07 (0.66–1.73)	.78
Medium-high	30 (35.7)		167 (37.3)			
Parental education						
No GCSEs	25 (29.1)		128 (26.8)		1.12 (0.67–1.85)	.67
GCSEs or higher	61 (70.9)		349 (73.2)			
ADHD diagnosis subtype						
DSM-IV combined	83 (67.5)		549 (72.1)		^c^	
DSM-IV inattentive	9 (7.3)		46 (6.0)		1.29 (0.61–2.74)	.50
DSM-IV hyperactive–impulsive	12 (9.8)		78 (10.2)		1.02 (0.53–1.95)	.96
DSM-III-R ADHD only^a^	16 (13.0)		69 (9.1)		1.53 (0.85–2.77)	.16
Life-time ADHD only^b^	3 (2.4)		20 (2.6)		0.99 (0.29–3.41)	.99
Separation anxiety (any symptoms)	41 (38.3)		164 (28.9)		1.53 (0.99–2.35)	.055
GAD (any symptoms)	15 (12.4)		56 (7.6)		1.73 (0.95–3.13)	.072
Social anxiety (any symptoms)	9 (7.5)		49 (6.7)		1.13 (0.54–2.37)	.75
Any anxiety (any symptoms)	46 (43.0)		219 (38.2)		1.22 (0.80–1.86)	.35
Depression (any symptoms)	80 (74.8)		451 (71.0)		1.21 (0.75–1.94)	.43
Core depression symptoms	17 (14.8)		71 (9.9)		1.59 (0.90–2.80)	.11
	**Mean (SE)**		**Mean (SE)**		**OR (LCI-UCI)**	***p***
Age at assessment	10.20 (0.26)		10.50 (0.10)		0.96 (0.90–1.03)	.30
Total IQ	84.20 (1.30)		84.30 (0.51)		1.00 (0.98–1.01)	.94
Inattentive ADHD symptoms	7.56 (0.15)		7.35 (0.06)		1.08 (0.96–1.23)	.21
Hyperactive–impulsive ADHD symptoms	7.62 (0.13)		7.75 (0.06)		0.95 (0.84–1.07)	.39
Total ADHD symptoms	15.20 (0.23)		15.10 (0.09)		1.01 (0.93–1.09)	.84

Abbreviations: GAD, generalized anxiety disorder; GCSE, General Certificate of Secondary Education.

a Child did not meet criteria for any DSM-IV subtype at assessment.

b Child met ADHD DSM-IV or DSM-III-R criteria previously but did not meet criteria at the time of assessment.

c Reference category.

**Table 2:** Testing for sex differences in psychiatric disorder genetic risk in the primary clinical sample of children with ADHD

PRS	Males	Females	OR (LCI-UCI)	*p*	*R* ^2^
Anxiety	760	123	1.00 (0.82–1.21)	.98	8.6E-07
MDD	762	123	1.12 (0.92–1.38)	.26	2.8E-03
ADHD	762	123	1.08 (0.90–1.29)	.44	1.1E-03
ASD	762	123	1.02 (0.85–1.24)	.80	1.2E-04
BD	762	123	1.22 (0.99–1.49)	.057	7.4E-03
Schizophrenia	760	123	1.18 (0.98–1.41)	.084	5.4E-03

Abbreviations: ADHD, attention deficit hyperactivity disorder; ASD, autism spectrum disorder; BD, bipolar disorder; MDD, major depressive disorder. Males are coded as “0” and females are coded as “1”; therefore OR > 1 indicates a higher PRS in females.

## Data Availability

The primary clinical sample genetic data & the PGC genetic data are available via application from: https://www.med.unc.edu/pgc/shared-methods/data-access-portal The ALSPAC data are available via application from: http://www.bristol.ac.uk/alspac/researchers/access/
