# Supplementary material for: Sex differences in anxiety and depression in children with attention deficit hyperactivity disorder: Investigating genetic liability and comorbidity
Source: Am J Med Genet B Neuropsychiatr Genet. Author manuscript; Available in PMC 2026 May 20. (PMC7619097; doi:10.1002/ajmg.b.32842)
Supplement: SM [file EMS213780-supplement-SM.docx]

# Supplemental Text

## Quality control of genetic data

DNA samples (from saliva or blood) were collected from children and where possible, both biological parents. A subset of the children were genotyped using the Illumina Human660W-Quad BeadChip for the purpose of a genome-wide association study (Stergiakouli et al., 2012). Children and parents were also genotyped using a customised version of the PsychChip. Quality control (QC) was performed within batch, as follows: SNPs were aligned to the Haplotype Reference Consortium (Loh et al., 2016) data using GenomeHarmoniser (Deelen et al., 2014), SNPs were removed if they had MAF<0.01, genotyping rate<0.95, or HWE p<10^-6^, and individuals were removed if they had missingness>0.05, sex discrepancy, or were duplicate samples. The data were merged with other samples on the same or equivalent platform and the QC parameters were reapplied, then the samples were imputed using the Michigan Imputation Server (using Eagle v2.4 for phasing, Minimac4 for imputation, and the HRC V1.1 imputation reference panel) (Das et al., 2016). After imputation, dosage data were converted to best guess genotype data using Plink2.

Post-imputation QC filters were genotype probability >0.9 per individual, missingness <0.03, MAF>0.01, HWE>10^-4^, and INFO/r^2^>0.8. Batches were merged, including only overlapping SNPs and excluding ambiguous (CT/AG) variants and SNPs with inconsistent alleles. Family relationships were confirmed using identity-by-descent (IBD) and Mendel analyses in PLINK. SNPs with >5 Mendel errors were excluded, and all remaining Mendel errors were set to missing. Samples that were not related as expected based on family structure were excluded. PCAiR (Conomos, Miller, & Thornton, 2015), a package that robustly estimates population structure while taking into account kinship information in the sample, was used to run a principal components analysis (PCA), on an LD-pruned set of common (MAF>0.05) markers and non-European samples were excluded, given that the discovery genome-wide association studies (GWAS) available were of European ancestries (A. R. Martin et al., 2019). A GWAS of batch was run on unrelated samples and SNPs associated with batch (p<0.01) were excluded. PCAiR was run again on the final set of markers to extract PCs to use as covariates. Only samples from children who passed all of the above QC were included in the study.

## PRS calculation

Polygenic risk scores (PRS) for common autosomal variants were derived in PLINK based on 6 large psychiatric disorder discovery GWAS of European ancestry, with no overlap with the target sample; for ADHD, the GWAS meta-analysis excluded the current samples. For each discovery GWAS, we selected common (MAF>0.05) variants that overlapped with the target data and performed linkage disequilibrium (LD) clumping in PLINK (--clump-kb 500 --clump-r2 0.2) to obtain an independent set of SNPs, while retaining the most significant SNP in each LD block. For schizophrenia PRS, we additionally excluded all variants in the extended MHC region (chromosome 6, base positions 25–35Mb) to avoid potential bias by extensive LD in this region. PRS were calculated for each individual by summing the number of alleles (weighted by the log of the odds ratio) across the set of SNPs in PLINK. We calculated PRS using 7 different p-value thresholds to select SNPs (p_T_<1, p_T_<0.5, p_T_<0.1, p_T_<0.05, p_T_<0.01, p_T_<0.001, p_T_<0.00001). For each discovery phenotype, we then performed PCA of the correlation matrix of these 7 PRS and extracted the first PC for analyses, following the PRS-PCA method, an approach that reduces overfitting and has been shown to maintain good power (Coombes, Ploner, Bergen, & Biernacka, 2020). The sign of the loadings of the PRS variables on the first PCs is arbitrary and therefore PRS-PCs that were negatively correlated with the raw PRS variables were inverted. For each of the 6 discovery phenotypes, the first PRS-PCs explained between 65.3–78.8% of the variation in the different p-value threshold PRS. The PRS-PCs were standardised using z-score transformations. 2 samples that were extreme outliers (PRS < -4SD) were excluded from the analyses using anxiety and schizophrenia PRS. Table S9 shows the correlations between the PRS-PC variable and the individual PRS based on different p-value thresholds, for anxiety and major depressive disorder.

## ALSPAC phenotypic variables and genetic data

ALSPAC originally recruited N=14,541 pregnant women resident in Avon, England, with expected delivery dates between April 1, 1991, and December 31, 1992. Of these, a total of N=13,988 children were alive at age 1 year (Boyd et al., 2013; Fraser et al., 2013). An additional sample of 913 children were enrolled after age 7, with a total of 14,901 children who were alive at 1 year of age. The study website contains details of all available data through a fully searchable data dictionary (<http://www.bris.ac.uk/alspac/researchers/data-access/data-dictionary/>). Ethical approval was obtained from the ALSPAC Ethics and Law Committee and Local Research Ethics Committees. Consent for biological samples has been collected in accordance with the Human Tissue Act (2004). Informed consent for the use of data collected via questionnaires and clinics was obtained from participants following the recommendations of the ALSPAC Ethics and Law Committee at the time.

Information on ADHD was available from the parent-completed Strengths and Difficulties Questionnaire (SDQ) (R. Goodman, 1997), which was completed at ages 4, 7, 8, 10, 12, and 13 years. The SDQ hyperactivity sub-scale includes 5 items related to ADHD (total score range: 0-10), with a recommended cut point of ≥8 for likely ADHD (R. Goodman, 1997).

Anxiety was assessed at multiple time points using a semi-structured interview, the Development and Well-Being Assessment (DAWBA) (R. Goodman, Ford, Richards, Gatward, & Meltzer, 2000). We focused on GAD, separation anxiety disorder, and social phobia at ages 7, 10, and 13 years (parent-rated), as well as GAD and social phobia/agoraphobia at age 15 years (self-rated). Algorithm-based DAWBA bands can predict the probability of a psychiatric disorder being present (A. Goodman, Heiervang, Collishaw, & Goodman, 2011). These bands have been well-validated in community samples of children, showing excellent agreement with clinician-rated diagnoses using a cut-off band of >50% probability of diagnosis and also a dose-response relationship for all bands (A. Goodman et al., 2011). To increase power and for the sake of comparability with our clinical ADHD sample, we defined anxiety symptom presence using a lower band (>15% probability of diagnosis) to better capture children with any anxiety symptoms, not just those with likely disorder. Binary variables were derived to indicate presence of any anxiety symptoms at any of the 4 time points and also for the 3 sub-types of anxiety.

ALSPAC children were genotyped using the Illumina HumanHap550 quad SNP genotyping platform. Children from triplet and quadruplet births were excluded and only one child from twin births was included in genotyping. Detailed QC procedures have been published previously (e.g. J. Martin, Hamshere, Stergiakouli, O’Donovan, & Thapar, 2014). PRS were derived using PRSice (Euesden, Lewis, & O’Reilly, 2015), using the same PLINK parameters as above and analyses used the first PRC-PCs for each discovery phenotype. The top 5 PCs from an ancestry-based sample PCA were included as covariates to account for population stratification.

**References**

Boyd, A., Golding, J., Macleod, J., Lawlor, D. A., Fraser, A., Henderson, J., … Davey Smith, G. (2013). Cohort Profile: the ’children of the 90s’--the index offspring of the Avon Longitudinal Study of Parents and Children. *International Journal of Epidemiology*, *42*(1), 111–127. https://doi.org/10.1093/ije/dys064

Conomos, M. P., Miller, M. B., & Thornton, T. A. (2015). Robust inference of population structure for ancestry prediction and correction of stratification in the presence of relatedness. *Genetic Epidemiology*, *39*(4), 276–293. https://doi.org/10.1002/gepi.21896

Coombes, B. J., Ploner, A., Bergen, S. E., & Biernacka, J. M. (2020). A principal component approach to improve association testing with polygenic risk scores. *Genetic Epidemiology*, *44*(7), gepi.22339. https://doi.org/10.1002/gepi.22339

Das, S., Forer, L., Schönherr, S., Sidore, C., Locke, A. E., Kwong, A., … Fuchsberger, C. (2016). Next-generation genotype imputation service and methods. *Nature Genetics*. https://doi.org/10.1038/ng.3656

Deelen, P., Bonder, M. J., Van Der Velde, K. J., Westra, H. J., Winder, E., Hendriksen, D., … Swertz, M. A. (2014). Genotype harmonizer: Automatic strand alignment and format conversion for genotype data integration. *BMC Research Notes*, *7*(1). https://doi.org/10.1186/1756-0500-7-901

Euesden, J., Lewis, C. M., & O’Reilly, P. F. (2015). PRSice: Polygenic Risk Score software. *Bioinformatics*. https://doi.org/10.1093/bioinformatics/btu848

Fraser, A., Macdonald-Wallis, C., Tilling, K., Boyd, A., Golding, J., Smith, G. D., … Lawlor, D. A. A. (2013). Cohort Profile: The Avon Longitudinal Study of Parents and Children: ALSPAC mothers cohort. *International Journal of Epidemiology*, *42*(1), 97–110. https://doi.org/10.1093/ije/dys066

Goodman, A., Heiervang, E., Collishaw, S., & Goodman, R. (2011). The ‘DAWBA bands’ as an ordered-categorical measure of child mental health: description and validation in British and Norwegian samples. *Social Psychiatry and Psychiatric Epidemiology*, *46*(6), 521–532. https://doi.org/10.1007/s00127-010-0219-x

Goodman, R. (1997). The Strengths and Difficulties Questionnaire: a research note. *Journal of Child Psychology and Psychiatry, and Allied Disciplines*, *38*(5), 581–586.

Goodman, R., Ford, T., Richards, H., Gatward, R., & Meltzer, H. (2000). The Development and Well-Being Assessment: Description and Initial Validation of an Integrated Assessment of Child and Adolescent Psychopathology. *Journal of Child Psychology and Psychiatry*, *41*(5), 645–655. https://doi.org/10.1111/j.1469-7610.2000.tb02345.x

Loh, P. R., Danecek, P., Palamara, P. F., Fuchsberger, C., Reshef, Y. A., Finucane, H. K., … Price, A. L. (2016). Reference-based phasing using the Haplotype Reference Consortium panel. *Nature Genetics*, *48*(11), 1443–1448. https://doi.org/10.1038/ng.3679

Martin, A. R., Kanai, M., Kamatani, Y., Okada, Y., Neale, B. M., & Daly, M. J. (2019). Clinical use of current polygenic risk scores may exacerbate health disparities. *Nature Genetics*. https://doi.org/10.1038/s41588-019-0379-x

Martin, J., Hamshere, M. L., Stergiakouli, E., O’Donovan, M. C., & Thapar, A. (2014). Genetic risk for attention-deficit/hyperactivity disorder contributes to neurodevelopmental traits in the general population. *Biological Psychiatry*, *76*(8), 664–671. https://doi.org/10.1016/j.biopsych.2014.02.013

Stergiakouli, E., Hamshere, M., Holmans, P., Langley, K., Zaharieva, I., Hawi, Z., … Thapar, A. (2012). Investigating the contribution of common genetic variants to the risk and pathogenesis of ADHD. *American Journal of Psychiatry*, *169*(2), 186–194.

# Supplementary Tables

# Table S1: Details of the DSM-IV anxiety and depression symptoms measured in the primary ADHD sample

| **Separation Anxiety Disorder of Childhood (DSM-IV 309.21)** | **Symptom presence, N(%)** |
| --- | --- |
| Distress at separation, coded as present if any of the following is present:   - Anticipatory distress - Withdrawal when attachment figure absent   Actual distress when attachment figure absent | 46 (5.3) |
| Worries about possible harm befalling attachment figures | 32 (4.6) |
| Worries about calamitous separation | 15 (2.2) |
| Worries about going to school, coded as present if any of the following is present:   - - Worries/anxiety about leaving home   - Fear when away of what will happen at home   - Stays at home some mornings (due to worry/anxiety)   - Child fails to reach or leaves school and returns home (due to worry/anxiety)   - Child fails to reach or leaves school and goes off alone (due to worry/anxiety)   Child fails to reach or leaves school and goes off with peers (due to worry/anxiety) | 75 (10.5) |
| Worries about sleeping, coded as present if any of the following is present:   - Reluctance to sleep alone - Sleeps with family member - Rising to check on family members - Avoidance of sleeping away from family | 107 (15.8) |
| Avoidance of being alone | 51 (7.4) |
| Separation dreams/nightmares | 25 (3.6) |
| Physical symptoms of separation | 79 (11.2) |
| **Any of the above 8 symptoms** | 205 (30.4) |
| **Generalised Anxiety Disorder (DSM-IV 300.02)** |  |
| Intrusive worries of 2 or more of the following types:   - Future events - Past behaviour - Performance - Self-consciousness - Appearance - Money - Other (worries/anxiety about leaving home, anticipatory fear of school,  fear when away of what will happen at home, hypochondriasis) | 71 (8.3) |
| **Social Anxiety Disorder (Social Phobia) (DSM-IV 300.23)** |  |
| Social anxiety or fear of activities in public | 44 (5.2) |
| Avoidance of social situations | 52 (6.1) |
| **Any of the above 2 symptoms** | 58 (6.8) |
| **Any of the above anxiety symptoms** | 265 (38.9) |
| **Depression (DSM-IV 296.2 – 296.3)** |  |
| Episode of depressed or irritable mood  (Period of 2 consecutive weeks where depressed mood was present for at least 8 days) | 58 (6.8) |
| Loss of interest / anhedonia | 40 (4.7) |
| **Any of the above 2 ‘core’ depression symptoms** | **88 (10.5)** |
| Reduced appetite / excessive appetite or weight loss / gain | 216 (24.9) |
| Insomnia / hypersomnia | 226 (26.0) |
| Subjective agitation / motor slowing | 27 (3.2) |
| Subjective anergia / tiredness / fatigability | 43 (5.0) |
| Pathological guilt / delusions of guilt / feelings of worthlessness | 297 (36.1) |
| Inefficient thinking / indecisiveness | 201 (23.8) |
| Thinking about death / suicidal thoughts / plans / attempts / behaviour without intent | 49 (8.0) |
| **Any of the above 9 depression symptoms** | **581 (75.7)** |

# Table S2: Association of polygenic risk scores (PRS) for anxiety disorders with anxiety symptoms and major depressive disorder (MDD) PRS with depression symptoms in children with clinically-diagnosed ADHD (primary sample), including results stratified by sex

| **Outcome** | **PRS** | **Males & females** | | | | **Males** | | | | **Females** | | | | **Interaction P** |
| --- | --- | --- | --- | --- | --- | --- | --- | --- | --- | --- | --- | --- | --- | --- |
|  |  | **N** | **OR (CIs)** | **P** | **R^2^** | **N** | **OR (CIs)** | **P** | **R^2^** | **N** | **OR (CIs)** | **P** | **R^2^** |  |
| **GAD** | Anxiety | 858 | 1.38 (1.09-1.76) | **0.0082** | 0.017 | 737 | 1.52 (1.16-2.00) | **0.0026** | 0.028 | 121 | 0.92 (0.51-1.67) | 0.79 | 0.001 | 0.14 |
| **Social anxiety** | Anxiety | 850 | 1.38 (1.06-1.81) | 0.017 | 0.017 | 730 | 1.54 (1.15-2.06) | **0.0040** | 0.029 | 120 | 0.74 (0.44-1.27) | 0.28 | 0.01 | **0.0049** |
| **Separation anxiety** | Anxiety | 672 | 0.99 (0.84-1.17) | 0.91 | 3E-05 | 565 | 1.07 (0.89-1.29) | 0.45 | 0.002 | 107 | 0.64 (0.41-1.00) | 0.052 | 0.041 | 0.020 |
| **Any anxiety** | Anxiety | 679 | 1.10 (0.94-1.28) | 0.24 | 0.003 | 572 | 1.21 (1.02-1.43) | 0.028 | 0.011 | 107 | 0.65 (0.41-1.03) | 0.065 | 0.047 | **0.0055** |
| **Core depression** | MDD | 836 | 1.00 (0.81-1.24) | 0.99 | 8E-07 | 721 | 0.89 (0.70-1.12) | 0.31 | 0.003 | 115 | 1.51 (0.93-2.46) | 0.095 | 0.03 | 0.033 |
| **Any depression** | MDD | 768 | 0.94 (0.78-1.12) | 0.47 | 0.001 | 656 | 0.97 (0.80-1.18) | 0.78 | 2E-04 | 112 | 0.77 (0.44-1.35) | 0.36 | 0.017 | 0.30 |

Bolded p-values show evidence of association after Bonferroni multiple testing correction (p<0.0083).

# Table S3: Association of polygenic risk scores (PRS) for anxiety disorders with anxiety symptoms in children with broadly-defined ADHD (in ALSPAC)

| **Outcome** | **Males & females** | | | | **Males** | | | | **Females** | | | | **Interaction P** |
| --- | --- | --- | --- | --- | --- | --- | --- | --- | --- | --- | --- | --- | --- |
|  | **N** | **OR (CIs)** | **P** | **R^2^** | **N** | **OR (CIs)** | **P** | **R^2^** | **N** | **OR (CIs)** | **P** | **R^2^** |  |
| **GAD** | 494 | 1.07 (0.89-1.29) | 0.48 | 0.0014 | 320 | 1.02 (0.80-1.30) | 0.86 | 1.4E-04 | 174 | 1.13 (0.83-1.53) | 0.45 | 4.4E-03 | 0.59 |
| **Social anxiety** | 438 | 1.01 (0.77-1.32) | 0.95 | 1.6E-05 | 286 | 0.94 (0.67-1.33) | 0.74 | 7.7E-04 | 152 | 1.13 (0.73-1.76) | 0.58 | 3.3E-03 | 0.51 |
| **Separation anxiety** | 547 | 1.26 (0.99-1.60) | 0.056 | 0.012 | 362 | 1.09 (0.81-1.47) | 0.56 | 0.0016 | 185 | 1.61 (1.07-2.42) | 0.023 | 0.05 | 0.14 |
| **Any anxiety** | 484 | 1.17 (0.98-1.40) | 0.083 | 0.0083 | 310 | 1.11 (0.88-1.39) | 0.38 | 0.0033 | 174 | 1.25 (0.93-1.69) | 0.14 | 0.016 | 0.46 |

ALSPAC: Avon Longitudinal Study of Parents and Children; GAD: Generalised Anxiety Disorder.

# Table S4: Exploratory analysis: association of polygenic risk scores (PRS) for ADHD with anxiety and depression symptoms in children with clinically-diagnosed ADHD (primary sample)

| **Outcome** | **PRS** | **Males & females** | | | |
| --- | --- | --- | --- | --- | --- |
|  |  | **N** | **OR (CIs)** | **P** | **R^2^** |
| **GAD** | Anxiety | 860 | 0.97 (0.75-1.26) | 0.83 | 1.4E-04 |
| **Social anxiety** | Anxiety | 852 | 1.15 (0.88-1.51) | 0.30 | 0.003 |
| **Separation anxiety** | Anxiety | 674 | 0.95 (0.80-1.12) | 0.53 | 8.3E-04 |
| **Any anxiety** | Anxiety | 681 | 0.99 (0.84-1.15) | 0.85 | 6.9E-05 |
| **Core depression** | MDD | 836 | 0.85 (0.69-1.06) | 0.15 | 0.005 |
| **Any depression** | MDD | 768 | 0.98 (0.82-1.18) | 0.85 | 8.5E-05 |

GAD: Generalised Anxiety Disorder. Bonferroni corrected p-value threshold of 0.0083.

# Table S5: Sensitivity analysis: testing for sex differences in psychiatric disorder polygenic risk scores (PRS) in the primary clinical sample of children with ADHD, stratifying the sample by age at the time of assessment

| **PRS** | **Young group (ages 5-10 years)** | | | | | **Older group (ages 11-18 years)** | | | | |
| --- | --- | --- | --- | --- | --- | --- | --- | --- | --- | --- |
|  | **Males** | **Females** | **OR (LCI-UCI)** | **P** | **R^2^** | **Males** | **Females** | **OR (LCI-UCI)** | **P** | **R^2^** |
| Anxiety | 398 | 72 | 0.97 (0.76-1.25) | 0.83 | 1.5E-04 | 364 | 51 | 1.18 (0.89-1.57) | 0.24 | 5.6E-03 |
| MDD | 398 | 72 | 0.86 (0.67-1.11) | 0.24 | 0.005 | 362 | 51 | 1.22 (0.90-1.66) | 0.21 | 0.008 |
| ADHD | 398 | 72 | 0.96 (0.71-1.29) | 0.78 | 3.6E-04 | 364 | 51 | 1.37 (1.04-1.80) | 0.026 | 0.02 |
| ASD | 398 | 72 | 1.24 (0.95-1.61) | 0.12 | 0.008 | 362 | 51 | 1.13 (0.87-1.48) | 0.36 | 0.003 |
| BD | 398 | 72 | 1.03 (0.80-1.34) | 0.81 | 2.1E-04 | 364 | 51 | 1.01 (0.77-1.34) | 0.92 | 3.9E-05 |
| Schizophrenia | 398 | 72 | 1.25 (0.95-1.63) | 0.11 | 0.01 | 364 | 51 | 1.15 (0.86-1.56) | 0.35 | 0.004 |

Major depressive disorder (MDD); ADHD: attention deficit hyperactivity disorder; ASD: autism spectrum disorder; BD: bipolar disorder. Bonferroni corrected p-value threshold of 0.0083. Males are coded as ‘0’ and females are coded as ‘1’; therefore OR>1 indicates a higher PRS in females.

# Table S6: Sensitivity analysis: association of polygenic risk scores (PRS) for anxiety disorders with anxiety symptoms and major depressive disorder (MDD) PRS with depression symptoms in children with ADHD, stratifying the sample by age at the time of assessment

| **Outcome** | **PRS** | **Young group (ages 5-10 years)** | | | | | **Older group (ages 11-18 years)** | | | | |
| --- | --- | --- | --- | --- | --- | --- | --- | --- | --- | --- | --- |
|  |  | **Males & females** | | | | **Interaction P** | **Males & females** | | | | **Interaction P** |
|  |  | **N** | **OR (CIs)** | **P** | **R^2^** |  | **N** | **OR (CIs)** | **P** | **R^2^** |  |
| **GAD** | Anxiety | 460 | 1.11 (0.74-1.67) | 0.60 | 0.002 | 0.98 | 398 | 1.61 (1.16-2.23) | 0.004 | 0.04 | 0.03 |
| **Social anxiety** | Anxiety | 452 | 1.28 (0.82-2.00) | 0.28 | 0.009 | 0.065 | 398 | 1.47 (1.05-2.06) | 0.024 | 0.03 | 0.014 |
| **Separation anxiety** | Anxiety | 328 | 0.75 (0.58-0.98) | 0.034 | 0.02 | 0.10 | 344 | 1.25 (0.99-1.57) | 0.066 | 0.01 | 0.12 |
| **Any anxiety** | Anxiety | 330 | 0.82 (0.64-1.04) | 0.11 | 0.01 | 0.22 | 349 | 1.43 (1.14-1.78) | 0.002 | 0.04 | 0.011 |
| **Core depression** | MDD | 444 | 1.35 (0.95-1.91) | 0.092 | 0.01 | 0.20 | 392 | 0.88 (0.68-1.14) | 0.33 | 0.004 | 0.29 |
| **Any depression** | MDD | 392 | 0.92 (0.73-1.17) | 0.51 | 0.002 | 0.24 | 376 | 0.96 (0.73-1.26) | 0.76 | 4.7E-04 | 0.42 |

GAD: Generalised Anxiety Disorder. Bonferroni corrected p-value threshold of 0.0083.

# Table S7: Sensitivity analysis: testing for sex differences in psychiatric disorder polygenic risk scores (PRS) in the primary clinical sample of children with ADHD, including only children who met DSM-IV criteria at the time of assessment

| **PRS** | **Males** | **Females** | **OR (LCI-UCI)** | **P** | **R^2^** |
| --- | --- | --- | --- | --- | --- |
| Anxiety | 671 | 104 | 1.01 (0.83-1.23) | 0.94 | 1.2E-05 |
| MDD | 673 | 104 | 1.09 (0.87-1.37) | 0.45 | 0.0015 |
| ADHD | 673 | 104 | 1.13 (0.92-1.38) | 0.23 | 0.003 |
| ASD | 673 | 104 | 1.00 (0.82-1.22) | 1.00 | 2.8E-08 |
| BD | 673 | 104 | 1.30 (1.03-1.63) | 0.025 | 0.013 |
| Schizophrenia | 671 | 104 | 1.23 (1.01-1.50) | 0.043 | 0.0086 |

Major depressive disorder (MDD); ADHD: attention deficit hyperactivity disorder; ASD: autism spectrum disorder; BD: bipolar disorder. Bonferroni corrected p-value threshold of 0.0083. Males are coded as ‘0’ and females are coded as ‘1’; therefore OR>1 indicates a higher PRS in females.

# Table S8: Sensitivity analysis: association of polygenic risk scores (PRS) for anxiety disorders with anxiety symptoms and major depressive disorder (MDD) PRS with depression symptoms in children with ADHD, including only children who met DSM-IV criteria at the time of assessment

| **Outcome** | **PRS** | **Males & females** | | | | **Interaction P** |
| --- | --- | --- | --- | --- | --- | --- |
|  |  | **N** | **OR (CIs)** | **P** | **R^2^** |  |
| **GAD** | Anxiety | 596 | 1.02 (0.85-1.22) | 0.86 | 7E-05 | 0.013 |
| **Social anxiety** | Anxiety | 754 | 1.34 (1.04-1.73) | 0.022 | 0.015 | 0.19 |
| **Separation anxiety** | Anxiety | 748 | 1.42 (1.08-1.88) | 0.013 | 0.02 | 0.012 |
| **Any anxiety** | Anxiety | 601 | 1.11 (0.94-1.31) | 0.21 | 0.004 | 0.006 |
| **Core depression** | MDD | 736 | 1.04 (0.83-1.30) | 0.74 | 3E-04 | 0.032 |
| **Any depression** | MDD | 679 | 0.94 (0.78-1.14) | 0.54 | 9E-04 | 0.24 |

GAD: Generalised Anxiety Disorder. Bonferroni corrected p-value threshold of 0.0083.

# Table S9: Correlations between the PRS-PC variables and the individual polygenic risk scores (PRS) based on different p-value thresholds, for anxiety and major depressive disorder (MDD) in the primary clinical sample of children with ADHD

| **Anxiety** | **PC1** | **p_T_ < 0.00001** | **p_T_ < 0.001** | **p_T_ < 0.01** | **p_T_ < 0.05** | **p_T_ < 0.1** | **p_T_ < 0.5** |
| --- | --- | --- | --- | --- | --- | --- | --- |
| **p_T_ < 0.00001** | 0.30 | 1.00 |  |  |  |  |  |
| **p_T_ < 0.001** | 0.60 | 0.37 | 1.00 |  |  |  |  |
| **p_T_ < 0.01** | 0.82 | 0.27 | 0.61 | 1.00 |  |  |  |
| **p_T_ < 0.05** | 0.93 | 0.22 | 0.48 | 0.77 | 1.00 |  |  |
| **p_T_ < 0.1** | 0.94 | 0.17 | 0.43 | 0.70 | 0.91 | 1.00 |  |
| **p_T_ < 0.5** | 0.92 | 0.13 | 0.38 | 0.62 | 0.82 | 0.90 | 1.00 |
| **p_T_ < 1** | 0.92 | 0.14 | 0.38 | 0.61 | 0.81 | 0.89 | 0.99 |
| **MDD** | **PC1** | **p_T_ < 0.00001** | **p_T_ < 0.001** | **p_T_ < 0.01** | **p_T_ < 0.05** | **p_T_ < 0.1** | **p_T_ < 0.5** |
| **p_T_ < 0.00001** | 0.42 | 1.00 |  |  |  |  |  |
| **p_T_ < 0.001** | 0.66 | 0.59 | 1.00 |  |  |  |  |
| **p_T_ < 0.01** | 0.83 | 0.39 | 0.67 | 1.00 |  |  |  |
| **p_T_ < 0.05** | 0.92 | 0.27 | 0.50 | 0.75 | 1.00 |  |  |
| **p_T_ < 0.1** | 0.93 | 0.23 | 0.46 | 0.68 | 0.91 | 1.00 |  |
| **p_T_ < 0.5** | 0.91 | 0.19 | 0.39 | 0.60 | 0.80 | 0.88 | 1.00 |
| **p_T_ < 1** | 0.90 | 0.18 | 0.38 | 0.60 | 0.79 | 0.87 | 0.99 |

# Supplementary Figures

**B**

**A**


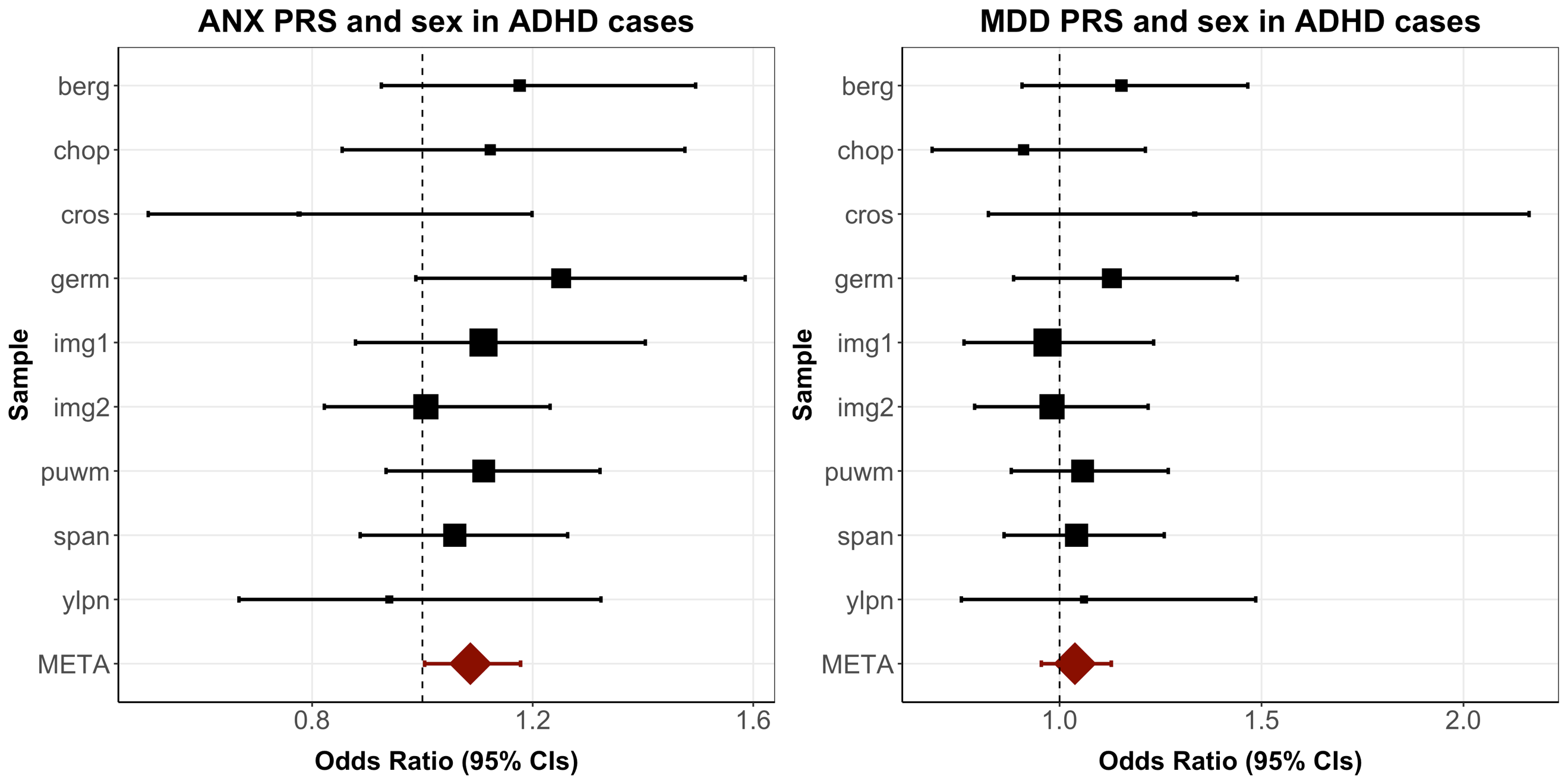


# Figure S1

Forest plot of meta-analysis results for 9 PGC ADHD studies for the association between polygenic risk scores (PRS) for a) anxiety disorder (ANX) and b) MDD with sex in ADHD cases (females coded as 1 and males coded as 0). All samples were of children, except that samples from Bergen (berg), Spain (span) & Yale-Penn (ylpn) were adults with ADHD.
