# Supplementary material for: Sex differences in anxiety and depression in children with attention deficit hyperactivity disorder: Investigating genetic liability and comorbidity
Source: Am J Med Genet B Neuropsychiatr Genet. Author manuscript; Available in PMC 2026 May 20. (PMC7619097; doi:10.1002/ajmg.b.32842)
Supplement: Appendix [file EMS213780-supplement-Appendix.pdf]

SUPPORTING INFORMATION

Additional supporting information may be found online in the Supporting Information section at the end of this article.

APPENDIX

Members of the Psychiatric Genomics Consortium ADHD Working Group

Ole A. Andreassen[1,2], Aurina Arnatkeviciute[3], Tobias Banaschewski[4], Mark Bellgrove[3], Anders Borglum[5,6,7], Jan Buitelaar[8], Christie Burton[9], Alice Charach[10], Elizabeth Corfield[11], Bru Cormand[12,13,14,15], Jennifer Crosbie[9], Ditte Demontis[5,6,7], Steve V. Faraone[16], Barbara Franke[17], Ian Gizer[18], Eugenio H. Grevet[19,20], Jan Haavik[21,22], Hakon Hakonarson[23,24], Catharina Hartman[25], Alexandra Havdahl[26,11,27], Ziarih Hawi[3], Johannes Hebebrand[28], Anke Hinney[28], Ridha Joober[29], Richard Karlsson Linnér[30,31], Marieke Klein[32], Jonna Kuntsi[33], Henrik Larsson[34,35], Klaus P. Lesch[36,37,38], Patrick W L. Leung[39], Calwing Liao[40], Sandra Loo[41], Sarah Medland[42], Alexandra Philipsen[43], Josep A. Ramos-Quiroga[44,45,46,47], Ted Reichborn-Kjennerud[48,49], Andreas Reif[50], Marta Ribases[44,45,46,51], Giovanni Salum[52], Russell Schachar[9], Sarojini M. Sengupta[29], Tim Silk[53,54], María Soler Artigas[44,45,46,51], Edmund Sonuga-Barke[55], Martin Tesli[11], Irwin Waldman[56], Thomas Werge[5,57,58,59], Stephanie H. Witt[60].

[1]NORMENT Center, Division of Mental Health and Addiction, Oslo University Hospital & Institute of Clinical Medicine, University of Oslo, Oslo, Norway; [2]KG Jebsen Centre for Neurodevelopmental Disorders, University of Oslo, Oslo, Norway; [3]Turner Institute for Brain and Mental Health, School of Psychological Sciences, Monash University, Melbourne, Australia; [4]Department of Child & Adolescent Psychiatry, Central Institute of Mental Health, Medical Faculty Mannheim/Heidelberg University; [5]The Lundbeck Foundation Initiative for Integrative Psychiatric Research, iPSYCH; [6]Department of Biomedicine, Aarhus University, Aarhus, Denmark; [7]Center for Genomics and Personalized Medicine, CGPM, Aarhus, Denmark; [8]Department of Cognitive Neuroscience, Donders Institute for Brain, Cognition and Behavior, Radboudumc, Nijmegen, The Netherlands; [9]Neurosciences and Mental Health, Hospital for Sick Children, Toronto, Ontario, Canada; [10]Hospital for Sick Children, Department of Psychiatry, University of Toronto, Toronto, Ontario, Canada; [11]Department of Mental Disorders, Norwegian Institute of Public Health, Oslo, Norway; [12]Department of Genetics, Microbiology, and Statistics, University of Barcelona, Catalonia, Spain; [13]Centro de Investigación Biomédica en Red de Enfermedades Raras (CIBERER), Spain; [14]Institut de Biomedicina de la Universitat de Barcelona (IBUB), Catalonia, Spain; [15]Institut de Recerca Sant Joan de Déu (IR-SJD), Esplugues de Llobregat, Barcelona, Catalonia Spain; [16]Departments of Psychiatry and Neuroscience and Physiology, Psychiatry Research Division, SUNY Upstate Medical University, Syracuse, New York, USA; [17]Departments of Human Genetics and Psychiatry, Donders Institute for Brain, Cognition, and Behavior, Radboud University Medical Center, Nijmegen, The Netherlands; [18]Department of Psychological and Brain Sciences, University of Missouri, Columbia, Missouri, USA; [19]Department of Psychiatry, Faculty of Medicine, Universidade Federal do Rio Grande do Sul; [20]Adult ADHD Outpatient Program (ProDAH), Clinical Research Center, Hospital de Clínicas de Porto Alegre, Rio Grande do Sul, Brazil; [21]KG Jebsen Centre for

Neuropsychiatric Disorders, Department of Biomedicine, University of Bergen, Bergen, Norway; [22]Division of Psychiatry, Haukeland University Hospital, Bergen, Norway; [23]The Center for Applied Genomics, Children's Hospital of Philadelphia, Philadelphia, Pennsylvania, USA; [24]Division of Human Genetics, Department of Pediatrics, The Perelman School of Medicine, University of Pennsylvania, Philadelphia, Pennsylvania, USA; [25]Department of Psychiatry, University of Groningen, University Medical Center Groningen, Groningen, The Netherlands; [26]Nic Waals Institute, Lovisenberg Diaconal Hospital, Oslo, Norway; [27]Department of Psychology, University of Oslo, Oslo, Norway; [28]Department of Child and Adolescent Psychiatry, University Hospital Essen, University of Duisburg-Essen, Duisburg, Germany; [29]Douglas Mental Health University Institute, Department of Psychiatry, McGill University, Montreal, Quebec, Canada; [30]Department of Economics, School of Business and Economics, Vrije Universiteit Amsterdam, Amsterdam, The Netherlands; [31]Autism and Developmental Medicine Institute, Geisinger, Lewisburg, Pennsylvania, USA; [32]Department of Psychiatry, University of California San Diego, La Jolla, California, USA; [33]Social, Genetic and Developmental Psychiatry Centre, Institute of Psychiatry, Psychology and Neuroscience, King's College London, London, UK; [34]School of Medical Sciences, Örebro University, Örebro, Sweden; [35]Department of Medical Epidemiology and Biostatistics, Karolinska Institutet, Stockholm, Sweden; [36]Division of Molecular Psychiatry, Center of Mental Health, University of Würzburg, Würzburg, Germany; [37]Laboratory of Psychiatric Neurobiology, Institute of Molecular Medicine, I.M. Sechenov First Moscow State Medical University, Moscow, Russia; [38]Department of Neuropsychology and Psychiatry, School for Mental Health and Neuroscience (MHeNS), Maastricht University, Maastricht, The Netherlands; [39]Department of Psychology, The Chinese University of Hong Kong, Shatin, NT, Hong Kong, China; [40]Department of Human Genetics, McGill University, Montreal, Quebec, Canada; [41]Semel Institute for Neuroscience and Human Behavior, UCLA David Geffen School of Medicine, Los Angeles, California, USA; [42]Psychiatric Genetics, QIMR Berghofer Medical Research Institute, Brisbane, QLD, Australia; [43]Department of Psychiatry and Psychotherapy, University Hospital Bonn, Bonn, Germany; [44]Department of Psychiatry, Hospital Universitari Vall d'Hebron, Barcelona, Catalonia, Spain; [45]Group of Psychiatry, Mental Health and Addictions, Psychiatric Genetics Unit, Vall d'Hebron Research Institute (VHIR), Barcelona, Catalonia, Spain; [46]Biomedical Network Research Centre on Mental Health (CIBERSAM), Barcelona, Catalonia, Spain; [47]Department of Psychiatry and Forensic Medicine, Universitat Autònoma de Barcelona, Barcelona, Catalonia, Spain; [48]Norwegian Institute of Public Health, Oslo, Norway; [49]Institute of Clinical Medicine, University of Oslo, Oslo, Norway; [50]Department for Psychiatry, Psychosomatic Medicine and Psychotherapy, University Hospital Frankfurt, Frankfurt am Main, Germany; [51]Department of Genetics, Microbiology, & Statistics, University of Barcelona, Barcelona, Spain.; [52]Department of Psychiatry, Universidade Federal do Rio Grande do Sul, Section on Negative Affect and Social Processes, Hospital de Clínicas de Porto Alegre; [53]School of Psychology, Deakin University, Melbourne, Australia; [54]Developmental Imaging, Murdoch Children's Research Institute, Melbourne, Australia; [55]School of Psychiatry, Institute of Psychiatry, Psychology & Neuroscience, King's College London, UK; [56]Department of Psychology, Emory University, Atlanta, Georgia, USA; [57]Institute of Biological Psychiatry, Mental Health Services, Copenhagen University Hospital, Copenhagen, Denmark; [58]Department of Clinical Medicine, University of Copenhagen, Copenhagen, Denmark; [59]Lundbeck Foundation Center for GeoGenetics, GLOBE Institute, University of Copenhagen, Copenhagen, Denmark; [60]Department of Genetic Epidemiology in Psychiatry, Central Institute of Mental Health, Medical Faculty Mannheim/Heidelberg University.
